# Development of Oxidized Polyvinyl Alcohol-Based Nerve Conduits Coupled with the Ciliary Neurotrophic Factor

**DOI:** 10.3390/ma12121996

**Published:** 2019-06-21

**Authors:** Andrea Porzionato, Silvia Barbon, Elena Stocco, Daniele Dalzoppo, Martina Contran, Enrico De Rose, Pier Paolo Parnigotto, Veronica Macchi, Claudio Grandi, Raffaele De Caro

**Affiliations:** 1 Department of Neurosciences, Section of Human Anatomy, University of Padova, Via A. Gabelli 65, 35121 Padova, Italy; andrea.porzionato@unipd.it (A.P.); elena.stocco@gmail.com (E.S.); martina.contran@gmail.com (M.C.); enrico.derose@studenti.unipd.it (E.D.R.); veronica.macchi@unipd.it (V.M.); raffaele.decaro@unipd.it (R.D.C.); 2 L.i.f.e.L.a.b. Program, Consorzio per la Ricerca Sanitaria (CORIS), Veneto Region, Via N. Giustiniani 2, 35128 Padova, Italy; 3 Department of Pharmaceutical and Pharmacological Sciences, University of Padova, Via F. Marzolo 5, 35128 Padova, Italy; dalzoppodaniele@gmail.com (D.D.); claudio.grandi@unipd.it (C.G.); 4 Foundation for Biology and Regenerative Medicine, Tissue Engineering and Signaling (T.E.S.) Onlus, 35030 Padua, Italy; pierpaolo.parnigotto@unipd.it

**Keywords:** nerve conduits, polyvinyl alcohol, chemical oxidation, ciliary neurotrophic factor, functionalization, drug delivery, bioactive scaffolds, peripheral nerve injury, neural regeneration

## Abstract

Functionalized synthetic conduits represent a promising strategy to enhance peripheral nerve regeneration by guiding axon growth while delivering therapeutic neurotrophic factors. In this work, hollow nerve conduits made of polyvinyl alcohol partially oxidized with bromine (OxPVA_Br_2_) and potassium permanganate (OxPVA_KMnO_4_) were investigated for their structural/biological properties and ability to absorb/release the ciliary neurotrophic factor (CNTF). Chemical oxidation enhanced water uptake capacity of the polymer, with maximum swelling index of 60.5% ± 2.5%, 71.3% ± 3.6% and 19.5% ± 4.0% for OxPVA_Br_2_, OxPVA_KMnO_4_ and PVA, respectively. Accordingly, hydrogel porosity increased from 15.27% ± 1.16% (PVA) to 62.71% ± 8.63% (OxPVA_Br_2_) or 77.50% ± 3.39% (OxPVA_KMnO_4_) after oxidation. Besides proving that oxidized PVA conduits exhibited mechanical resistance and a suture holding ability, they did not exert a cytotoxic effect on SH-SY5Y and Schwann cells and biodegraded over time when subjected to enzymatic digestion, functionalization with CNTF was performed. Interestingly, higher amounts of neurotrophic factor were detected in the lumen of OxPVA_Br_2_ (0.22 ± 0.029 µg) and OxPVA_KMnO_4_ (0.29 ± 0.033 µg) guides rather than PVA (0.11 ± 0.021 µg) tubular scaffolds. In conclusion, we defined a promising technology to obtain drug delivery conduits based on functionalizable oxidized PVA hydrogels.

## 1. Introduction

The clinical management of peripheral nerve injuries (PNI) is still far from ideal, prompting both surgeons and researchers to investigate new intervention strategies [1]. Nerve autograft remains the current gold standard treatment for bridging gaps (>30 mm) between the proximal and the distal nerve stumps and promoting nerve regeneration [2]. Nevertheless, it frequently results into unsatisfactory outcomes, being tissue scarring, insufficient length of nerve graft, formation of neuroma and the possible loss of sensation/function at the donor site the main issues that need to be addressed [3,4,5]. 

Nerve tissue engineering-based efforts are offering novel and promising opportunities to overcome autografting limitation by the development of nerve guidance conduits [6,7]. These function as tubular structures specifically designed to connect distal and proximal stumps of a sectioned nerve, meanwhile acting as a guide for axon regeneration along the proximal-distal axis, as well as a barrier against the ingrowth of cicatricial tissue [8,9]. To date, both synthetic polymers (i.e., poly-L-lactide acid (PLLA), polycaprolactone (PCL)), and natural biomaterials (i.e., collagen, fibrin) have been tested for nerve conduits fabrication [10,11]. Among these, synthetically based hydrogels seem to assure for better control and customization of biochemical/mechanical properties, while mitigating immune responses, which could lead to graft rejection [4].

Recently, we introduced for the first time the use of partially oxidized polyvinyl alcohol as an innovative material to prepare biocompatible synthetic prostheses for tissue engineering [12]. Protein loading ability and in vivo biodegradability of the original polymer could be efficiently improved by chemical oxidation with potassium permanganate. Taking advantage of implemented biological features of oxidized PVA, we reported the fabrication of bioabsorbable tubular hydrogel scaffolds, which turned out to be potentially effective candidates for guiding nerve regeneration [13].

Despite promising results, it has been recognized that providing conduits with adequate structural cues may be insufficient for achieving satisfactory regeneration, particularly in nerve resections with a loss of substance [4,5,6,7,8,9,10,11,12,13,14,15,16]. Advances in nerve conduit technology could be represented by the fabrication of neuroguides, which not only provide physical nerve guidance, but also allow for the diffusion of growth factors (GFs) and nutrients to ensure axon growth and Schwann cell survival/proliferation [8,17]. Thus, nerve guide functionalization with key neurotrophic factors has been recently emerged as a successful strategy to enhance neurogenic potential of synthetic tubular scaffolds [16]. In this context, drug delivery nerve guides were successfully fabricated by coupling ciliary neurotrophic factor (CNTF) [18], nerve growth factor (NGF) [16,19,20,21,22,23,24], brain-derived neurotrophic factor (BDNF) [21] or glial derived neurotrophic factor (GDNF) [20] to synthetic scaffolds based on poly{(lactic acid)-co-[(glycolic acid)-alt-(L-lysine)]} (PLGL) [19], poly(ethylene oxide) (PEO) [21], poly lactic-co-glycolic acid (PLGA) [16,18,22], poly(ester urethane) [20] and PCL [23,24].

Given our previous experience on (i) the fabrication of oxidized PVA-based nerve conduits [13] and (ii) the study of CNTF as a neuroprotective molecule with potential therapeutic benefits for PNI repair [25], we evaluated the functionalization with CNTF of tubular hydrogel scaffolds made of PVA oxidized with a novel method, based on the use of elementary bromine (OxPVA_Br_2_ scaffolds). This halogen could represent a less aggressive oxidation agent, which could better prevent molecular weight alterations of the polymer upon oxidation, providing a valid alternative to potassium permanganate to obtain more biodegradable PVA hydrogels with implemented protein loading and release capacity. Thus, considering the comparison with nerve guides made of neat PVA and PVA oxidized with potassium permanganate (OxPVA_KMnO_4_), this preliminary in vitro study investigated the ultrastructural morphology of OxPVA_Br_2_ scaffolds, their swelling behavior, porosity, mechanical features, cytotoxicity and in vitro biodegradation. Noteworthy, their capacity of acting as drug delivery systems by absorbing and releasing CNTF was also defined.

## 2. Materials and Methods

### 2.1. Reagents

Polyvinyl alcohol (molecular weight (MW) 124,000–184,000 Da), 70% hydrochloric acid, acetonitrile, elementary bromine, perchloric acid, sodium bicarbonate, bovine pancreatic trypsin, poly-L-lysine, Dulbecco’s Modified Eagle Medium/Nutrient Mixture F-12, Dulbecco’s Modified Eagle Medium (DMEM), fetal bovine serum, penicillin/streptomycin solution, gentamicin solution, Heregulin β1, Forskolin, ethylenediaminetetraacetic acid, [3-(4,5-dimethylthiazol-2-yl)-2,5-dimethyltetrazolium bromide] and dialysis membranes (cut-off 8000 Da) were purchased from Sigma-Aldrich (S. Louis, Missouri, USA). Potassium permanganate, ascorbic acid, acetic acid, 2-propanol, 100% ethanol, sodium iodide, sodium cyanoborohydride and dimethyl sulfoxide were purchased from Carlo Erba (Milan, Italy).

### 2.2. Preparation of PVA and Oxidized PVA Solutions

For the preparation of a 16% neat PVA solution, a pre-weighed quantity of polymer powder was suspended into milliQ water and heated for 48 h at 100 °C under stirring until complete dissolution [12]. Partial oxidation of PVA was performed by using two different oxidizing agents: Potassium permanganate (KMnO_4_) in dilute perchloric acid (HClO_4_) and bromine (Br_2_) in sodium bicarbonate (NaHCO_3_) buffer. The polymer solution was treated with the stoichiometric quantity of the oxidative agent sufficient to oxidize the 1% of secondary alcoholic groups to carbonyl groups.

OxPVA_KMnO_4_ (1% oxidized PVA with potassium permanganate) was prepared according to Stocco and Collaborators [12]. Briefly, 10 g of PVA were added to 200 g of milliQ water and heated in a boiling water bath for 60 min under agitation. After that, the polymer solution was cooled at 37 °C and treated with 156 mg of KMnO_4_ and 1.60 g of 70% perchloric acid (w/w) in 10 mL of milliQ water for 60 min till complete discoloration. To remove any residual of the oxidative process, the resulting solution was extensively dialyzed against water using a membrane with 8000 Da cut-off and finally lyophilized for long-term storage.

In parallel, OxPVA_Br_2_ (1% oxidized PVA with bromine) was obtained by dissolving 5.05 g of PVA powder into 100 g of milliQ water and heating the solution in a boiling water bath for 60 min over a magnetic stirrer. After the solution was cooled at 15 °C, 264 mg of NaHCO_3_ were added and mixed until complete dissolution. Then, 66 μL of Br_2_ in 11 mL of milliQ water were added and left to react for 4 h at 15–18 °C until a complete clear solution was obtained. Finally, the resulting solution was dialyzed and lyophilized as previously described.

### 2.3. Swelling Behavior of PVA-Based Hydrogel

Neat PVA and oxidized PVA disks (diameter: 7 mm; thickness: 2 mm) were prepared as described by Stocco and Collaborators [12] and separately immersed in 1 mL phosphate buffer solution (PBS) on a 24-well plate (Corning,) at room temperature (RT) for a total time of 600 h. Every 48 h, PBS excess was removed by wiping the tubes with filter paper and the samples were weighed by an analytical balance (mod.E/50 macro/semi-micro, Gibertini, Milan, Italy). The swelling ratio was calculated using the following equation:

(1) $$\mathrm{Swelling}\;\left(\%\right)=\frac{W_{s}-W_{d}}{W_{d}}\times 100,$$

where W_s_ is the weight of the swollen scaffold and W_d_ is the weight of the dry scaffold.

### 2.4. Porosity Measurements

PVA and oxidized PVA porosity was evaluated by the solvent replacement method [26]. Pre-weighted dried hydrogels were immersed in PBS for 48 h and then weighed again, after removing the excess of PBS on their surface. The porosity was calculated from the following equation:

(2) $$\mathrm{Porosity}\;\left(\%\right)=\frac{W_{s}-W_{d}}{\rho V}\times 100,$$

where W_d_ and W_s_ are the weight of hydrogel before and after the immersion in PBS, respectively; ρ is the density of PBS and V is the volume of the hydrogel.

### 2.5. Fabrication of Nerve Guidance Conduits

Tubular scaffolds of PVA, OxPVA_KMnO_4_ and OxPVA_Br_2_ were prepared through the injection molding-technique [13], starting from water solutions containing 16% (w/w) of each polymer obtained heating in a sealed vial the components at 110 °C and mixing for 2 h by several inversions. Each tubular scaffold was then assembled by aspiring the hot solution in a stainless-steel cylinder having an internal diameter of 2.1 mm and length of 5 cm, followed by coaxial introduction of a stainless-steel plunger with external diameter of 1 mm and length of 10 cm. Finally, the cross-linking of the polymer was performed by freeze-thawing process consisting of seven cycles of cooling at −20 °C for 24 h and thawing at −2.5 °C for 24 h. After removal from the molder, the nerve guides were kept at −20 °C until use.

### 2.6. Morphological Characterization

Ultrastructural analysis of PVA-based nerve guides was performed by scanning electron microscopy (SEM). Samples were dehydrated through a series of graded ethanols, exposed to critical-point drying and gold sputtering and finally observed. Images were taken using a JSM-6490 scanning electron microscope (Jeol USA, Peabody, MA, USA).

### 2.7. Mechanical Properties and Suture Test

A preliminary demonstration of flexibility, elasticity and mechanical strength of PVA- and oxidized PVA-based nerve guides was performed by means of suture tests using Nylon 8–0 sutures. A microsurgeon specialized in peripheral nerve reconstruction performed suture tests by passing the needle perpendicular to the surface of the conduit wall and exerting some tensile force to evaluate the mechanical resistance to the suture. Furthermore, a microsurgical anastomosis between two conduits was performed to test the material capacity to hold the suture knots.

### 2.8. In Vitro Cytotoxicity Assay

#### 2.8.1. Cell Cultures

Neat and oxidized PVA cytotoxic effects were evaluated on a) the human neuroblastoma cell line SH-SY5Y (ATCC, Manassas, VA, USA) and b) primary cultures of Schwann cells isolated from a rat sciatic nerve according to a published protocol [27,28]. SH-SY5Y cells were cultured in 25 cm^2^-flasks (Corning, NY, USA) using DMEM/F-12 (Dulbecco’s Modified Eagle Medium/Nutrient Mixture F-12) supplemented with 15% fetal bovine serum (FBS) and 1% penicillin (10,000 U/mL)/streptomycin (10 mg/mL) solution. In parallel, Schwann cells were cultured in 25 cm^2^-flasks on poly-L-lysine coating, using DMEM (Dulbecco’s Modified Eagle Medium) supplemented with 10% FBS, 50 μg/mL gentamicin solution, 1 nM Heregulin β1 and 1 nM Forskolin. Cultures were maintained at 37 °C, 95% relative humidity and 5% CO_2_ changing medium every other day until the time of use. SH-SY5Y cells of passage 35 and Schwann cells of passage 3 were used in the extract cytotoxicity test.

#### 2.8.2. Extract Test

The cytotoxicity of any leachable by-products from neat and oxidized PVA was assessed by the following described extract test. In short, neat PVA, OxPVA_KMnO_4_ and OxPVA_Br_2_ tubes (diameter: 1 mm; length: 10 mm) were incubated for 24 h into SH-SY5Y or Swann cell culture medium (100 mg/mL), at 37 °C. In parallel, cells at 80%–90% confluence were harvested by treatment with 0.025% ethylenediaminetetraacetic acid (EDTA) and 0.25% trypsin in PBS and seeded at high density (60,000 cells/cm^2^) on 96-well culture plates (Corning). After 24 h from seeding, the cell culture medium was removed and replaced with the extract medium. As positive (cytotoxic) control, cells were incubated in culture medium added with 50% dimethyl sulfoxide (DMSO; Sigma-Aldrich, S. Louis, Missouri, USA), whereas the negative control was represented by untreated cultures. Treated and control samples were incubated for 24 h at 37 °C, 95% relative humidity and 5% CO_2_ and the effect of extract medium on cell survival was then evaluated by the MTT [3-(4,5-dimethylthiazol-2-yl)-2,5-dimethyltetrazolium bromide] assay.

#### 2.8.3. MTT Assay

The viability of cells after incubation with PVA and oxidized PVA extracts was assessed by 3-(4,5-dimethylthiazol-2-yl)-2,5-dimethyltetrazolium bromide (MTT) assay [29]. Treated and untreated SH-SY5Y and Schwann cells were incubated with (a) 0.5 mg/mL MTT (Sigma-Aldrich, S. Louis, Missouri, USA) for 4 h and (b) 2-propanol acid (0.04 M HCl in 2-propanol; Carlo Erba, Milan, Italy) for 15 min to dissolve formazan precipitates. Finally, the optical density was measured at 570 nm by using a Microplate auto reader VICTOR3™ (PerkinElmer, Waltham, MA, USA). Results were expressed as relative cell viability measured in treated samples in comparison with the untreated control (100%).

### 2.9. In Vitro Biodegradation

Enzymatic degradation of neat and oxidized PVA nerve conduits was investigated by incubating samples in a 5 mg/mL collagenase B (Roche, Basel, Switzerland) solution and monitoring the weight loss over time, up to 14 days (d). To mimic physiological conditions, collagenase was dissolved in human plasma and samples were incubated at 37 °C. Nerve conduits were weighed at well-defined time points (1, 3, 5, 8 and 14 d), after being extracted from the collagenase solution and dried to remove solvent excess. At each time point (*t*), weight loss was calculated according to the following formula:

(3) $$\mathrm{Weight}\;\mathrm{loss}\;\left(\%\right)=\frac{W_{i}-W_{t}}{W_{t}}\times 100,$$

where W_i_ represents the initial weight and W_t_ represents the weight of the sample after the time point *t* [30].

### 2.10. Preparation of Functionalized Nerve Guidance Conduits

#### 2.10.1. Coupling CNTF to the Nerve Guides

Nerve guides made of PVA, OxPVA_KMnO_4_ and OxPVA_Br_2_ (diameter: 1 mm; length: 10 mm) were rinsed with PBS and filled with a 0.16 mg/mL CNTF (PeproTech, London, UK) solution in PBS pH 7.4 containing 3 mg/mL sodium cyanoborohydride. The concentration of CNTF for functionalization was chosen to prevent the precipitation of the growth factor in the solution, which we observed to occur when higher concentrations were tested. After 2 h at 16 °C the initial solution was discarded and substituted with a fresh one, continuing the coupling for further 4 h at 16°C. After that the tubes were rinsed internally five times with PBS, for 5 min each time.

#### 2.10.2. Digestion and Recovery of CNTF Tryptic Peptides

To recover absorbed CNTF, nerve guides were filled with a few microliters of a solution containing 0.40 mg/mL trypsin in PBS, kept at 16 °C for 15 min and then squeezed to recover all the inside solution in 500 μL Eppendorf tubes. After 5 min, further CNTF digestion was completely blocked with 50 μL of pure acetic acid and the solutions were lyophilized.

#### 2.10.3. Ultra High-Performance Liquid Chromatography-Mass Spectrometry (UHPLC-MS) Analysis of CNTF Tryptic Peptides

Tryptic peptides were separated on a Vyadc C18 (diameter: 1 mm; length: 15 cm) employing a linear acetonitrile gradient from 2% to 60% in 15 min at a flow rate of 50 µL/min. Both eluent, water and acetonitrile contained 0.1% formic acid and were pumped by an ultra high-performance liquid chromatography (UHPLC) Infinity 1290 (Agilent, Santa Clara, CA, USA) connected to a mass spectrometer XEVO G2S (Waters, Milford, MA, USA). Each sample of dry tryptic peptides was reconstituted with 15 μL of acetic acid and 15 μL of water and 7 μL of solution were injected for analysis. The mass spectrometer was previously calibrated with sodium iodide in m/z range 50–2000 and data accuracy was maintained by continuous acquisition of signal reference made of Leu-enkephalin (m/z = +556.2771). Quantitation of the amount of CNTF digested from the neuroguide internal lumen was obtained comparing the intensities of the ions signal from each sample with the intensities obtained by the peptides derived from complete digestion of CNTF (0.094 µg).

### 2.11. Statistical Analysis

For all experiments, data were expressed as mean ± standard deviation of three different replicates. Statistical analysis was performed by one-way analysis of variance (ANOVA) and Dunnett T3 multiple comparison test. *p* < 0.05 was considered to be statistically significant. Statistical calculations were carried out by Prism 8.1.0 (GraphPad Software, San Diego, CA, USA).

## 3. Results

### 3.1. Swelling Kinetics of PVA-Based Hydrogels

**Figure 1:**
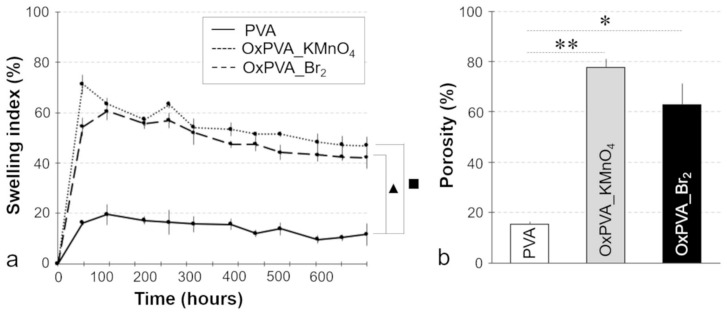
Swelling behavior and porosity of PVA-based hydrogels. Effect of chemical oxidation on PVA swelling kinetics (**a**) and porosity (**b**). The experiments were repeated on three samples of each hydrogel at all time points. Response is the average swelling/porosity of replicates ± standard deviation. (■: *p* < 0.05, OxPVA_KMnO_4_ vs. PVA; ▲: *p* < 0.05, OxPVA_Br_2_ vs. PVA; **: *p* < 0.01, OxPVA_KMnO_4_ vs. PVA; *: *p* < 0.05, OxPVA_Br_2_ vs. PVA).

The swelling index is directly proportional to the amount of water imbibed within a hydrogel, which influences the diffusional properties of a solute within the hydrogel itself [31]. The impact of Br_2_-mediated oxidation on polymer swelling properties was assessed through the comparison with non-modified PVA and PVA oxidized with KMnO_4_, which is known to acquire better absorption capacity due to chemical modification [12]. The PVA-based hydrogel swelling kinetics was studied by measuring the degree of swelling with time (Figure 1a). Accordingly, the maximum water retention capacity of OxPVA_Br_2_ hydrogel was 60.5% ± 2.5% of the dried weight, reached at 96 h of incubation. In parallel, maximum swelling index for neat PVA and OxPVA_KMnO_4_ was 19.5% ± 4.0% at 96 h and 71.3% ± 3.6% at 48 h, respectively. As previously observed for OxPVA_KMnO_4_, the general trend for OxPVA_Br_2_ is an enhancement of swelling index due to chemical modification in comparison with non-modified hydrogel. Comparing the two types of oxidized polymers, swelling index was observed to be not statistically different for OxPVA_Br_2_ vs. OxPVA_KMnO_4_ during the whole incubation period. Conversely, significant differences (*p* < 0.05) in swelling behavior were observed at all time points considering OxPVA_Br_2_ vs. PVA and OxPVA_KMnO_4_ vs. PVA.

### 3.2. Hydrogel Porosity

The absorption capacity of a hydrogel can be attributed to its grade of porosity, as more pores would enhance the uptake of water during swelling in comparison with less porous materials [26]. Thus, porosity of neat and oxidized PVA was measured and compared, as reported in Figure 1b. In accordance with swelling behavior, PVA porosity (15.27% ± 1.16%) increased after oxidation treatment, with no significant differences between OxPVA_Br_2_ (62.71% ± 8.63%) and OxPVA_KMnO_4_ (77.50% ± 3.39%) porosity.

### 3.3. Morphological Characterization of Nerve Guidance Conduits

**Figure 2:**
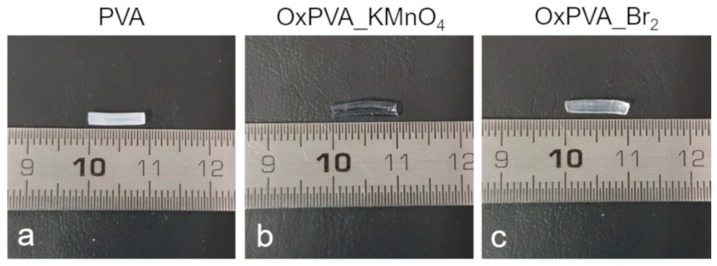
Tubular scaffolds morphology. Gross appearance of cross-linked PVA (**a**), OxPVA_KMnO_4_ (**b**) and OxPVA_Br_2_ (**c**) nerve guidance conduits manufactured according to the injection molding-technique.

After nerve guide manufacture, their gross appearance and ultrastructural morphology were characterized. The physical appearance of the PVA-based hollow conduits is shown in Figure 2 and results to be highly transparent as typical for hydrogel scaffolds. An augmented transparency of the material was detectable after oxidation, OxPVA_KMnO_4_ conduits appearing more transparent than OxPVA_Br_2_ guides. The lumen was clearly recognizable in all conduits.

**Figure 3:**
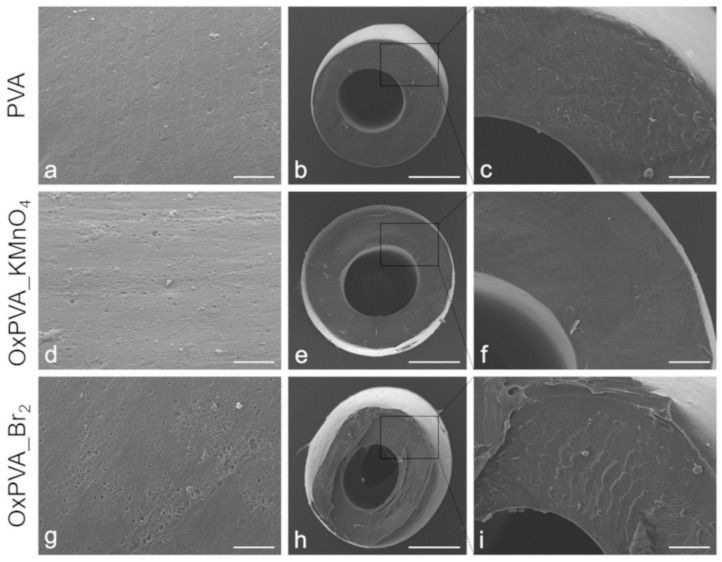
Ultrastructural analysis. SEM micrographs of crosslinked PVA-based nerve guidance conduits. (**a**,**d**,**g**) Side view and (**b**,**c**,**e**,**f**,**h**,**i**) transversal cross-sections of tubular scaffolds. Scale bars: (**a**,**d**,**g**) 10 µm; (**b**,**e**,**h**) 500 µm; (**c**,**f**,**i**) 100 µm.

The surface ultrastructure of nerve guides was analyzed by SEM, as represented in Figure 3. Native PVA conduits exhibit a smoother surface compared to oxidized PVA-based samples, with an increased rugosity detectable in OxPVA_Br_2_ nerve guides. Transverse cross-sections of tubular scaffolds demonstrate a certain regularity of the wall conduits.

### 3.4. Mechanical Properties and Suture Test

In the perspective of in vivo nerve graft studies, mechanical consistency, flexibility and suture holding ability appear to be crucial properties for handling and implantation of conduits and axonal growth promotion. Even more important, tubular conduits need to display adequate mechanical features to respond to limb flexion and withstand the pressure of surrounding tissues (i.e., muscles and bones).

**Figure 4:**
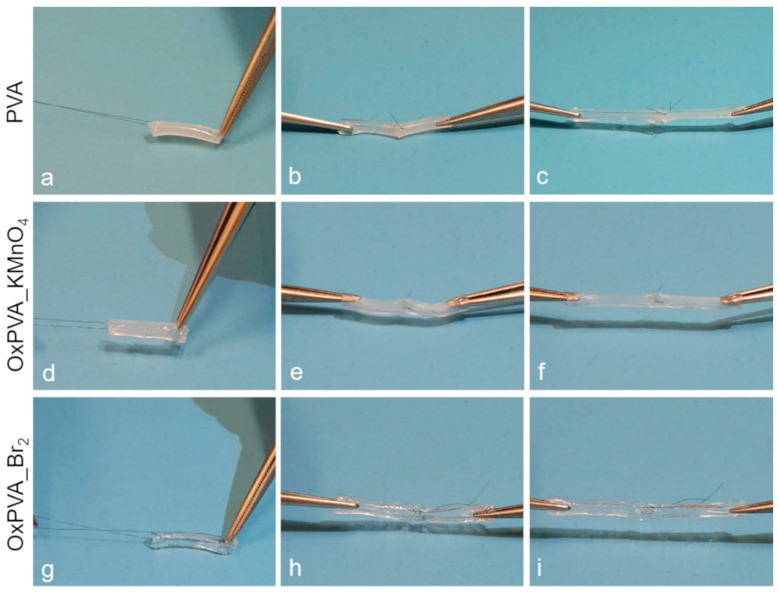
Suture test. Neat and oxidized PVA tubular scaffolds were tested for suture holding ability by passing the needle perpendicular to the surface of the conduit wall and exerting some tensile force (**a**,**d**,**g**). Furthermore, a microsurgical anastomosis between two conduits was performed (**b**,**e**,**h**) and the material capacity to hold the suture knots was assessed by stretching the sutured conduits (**c**,**f**,**i**).

The mechanical behavior and suture holding ability of PVA, OxPVA_KMnO_4_ and OxPVA_Br_2_ nerve guidance conduits are demonstrated by Videos S1–S3, respectively. All the conduits were easy to handle and exhibited adequate consistency to be sutured. They also showed to be mechanically robust, with excellent properties of elasticity/flexibility. Neuroguides did not collapse with the needle passage, held sutures well during microsurgical anastomosis and could be stretched without ruptures of the conduit wall (Figure 4). Among the three types of scaffolds, OxPVA_Br_2_ samples seemed to be the less consistent, but they still showed mechanical resistance and high flexibility.

### 3.5. Cytotoxicity Assay

**Figure 5:**
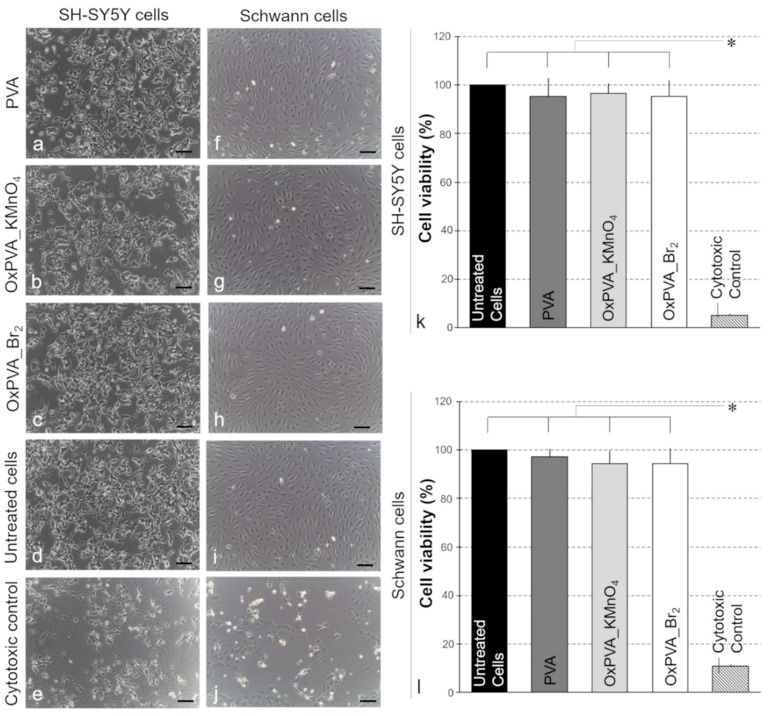
Cytotoxicity test. (**a**–**j**) Morphological analysis by optical microscopy of SH-SY5Y (**a**–**e**) and Schwann cell (**f**–**j**) cultures after treatment with native and oxidized PVA-conditioned media. (**k**,**l**) Cell viability of SH-SY5Y (**k**) and Schwann cell (**l**) cultures after treatment with native and oxidized PVA-conditioned media. Data are the average of three different experiments ± standard deviation and results are presented as percentage of living cells compared to the untreated control (*: *p* < 0.01). Scale bars: 100 µm.

The cytotoxicity of neat and oxidized PVA conduits was investigated using the extract test. As demonstrated by Figure 5a–j, no significant morphological changes were observed in both SH-SY5Y and Schwann cell populations that were incubated with polymer-conditioned media. As evidenced by MTT assay (Figure 5k,l), there was no statistical difference in the cell viability of SH-SY5Y and Schwann cell populations cultured with neat and oxidized PVA-conditioned media and the blank culture medium. Conversely, a significant preservation of cell viability and proliferation was observed in treated samples compared to the cytotoxic control, 50% DMSO. Setting to 100% the viability of untreated cells, the relative cell viability was 95.2% ± 7.7% (PVA), 96.6% ± 4.1% (OxPVA_KMnO_4_), 95.3% ± 6.6% (Br_2_) and 5.0% ± 0.5% (DMSO) for SH-SY5Y cells. In parallel, the relative cell viability of Schwann cell cultures was 97.2 ± 3.2% (PVA), 94.2% ± 5.4% (OxPVA_KMnO_4_), 94.5% ± 6.2% (Br_2_) and 10.8% ± 0.7% (DMSO). 

### 3.6. Enzymatic Degradation of Nerve Conduits

**Figure 6:**
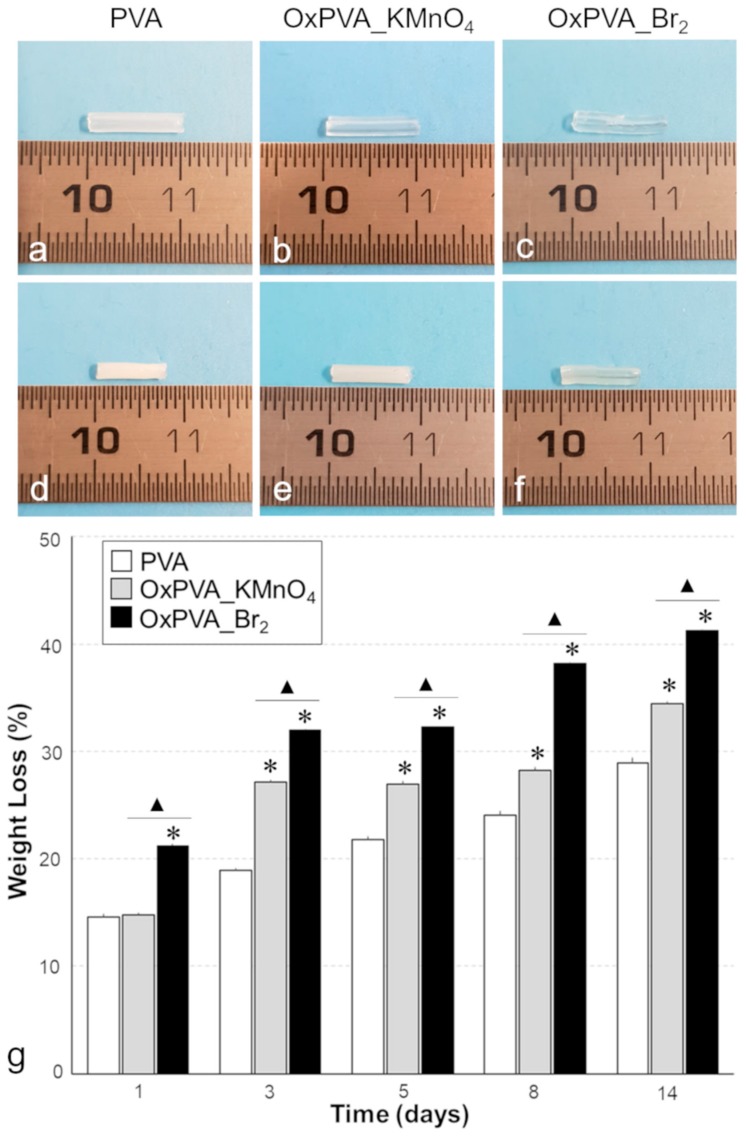
Enzymatic degradation. (**a**–**f**) Gross appearance of neat and oxidized PVA nerve conduits soon after manufacture (**a**,**b**,**c**) and after 14 days of incubation in human plasma + collagenase B solution (**d**,**e**,**f**). (**g**) Sample weight loss measured at 1, 3, 5, 8 and 14 days of incubation in enzymatic solution (*: *p* < 0.01, OxPVA_KMnO_4_ or OxPVA_Br_2_ vs. PVA; ▲: *p* < 0.01, OxPVA_KMnO_4_ vs. OxPVA_Br_2_).

Biodegradability represents a fundamental property for polymeric scaffolds intended for tissue engineering applications. To compare the biodegradability of PVA, OxPVA_KMnO_4_ and OxPVA_Br_2_ tubular scaffolds, weight loss (%) after enzymatic degradation was measured up to 14 days. When macroscopically analyzed at the end of the incubation period, the tubular scaffolds appeared to be smaller and more opaque than before treatment (Figure 6a–f). All nerve conduits showed increasing weight loss over time, suggesting sample progressive degradation. Higher weight loss rates were registered for OxPVA_KMnO_4_ (from 14.8% at day 1 to 34.4% at day 14) and OxPVA_Br_2_ (from 21.2% to 41.25%) conduits in comparison with PVA-based scaffolds (from 14.6% to 28.9%; Figure 6g). Remarkably, bromine-mediated oxidation seemed to significantly increase sensibility of polymeric scaffolds to enzymatic degradation in comparison with KMnO_4_-based modification.

### 3.7. Release of CNTF from Functionalized Nerve Guides

**Figure 7:**
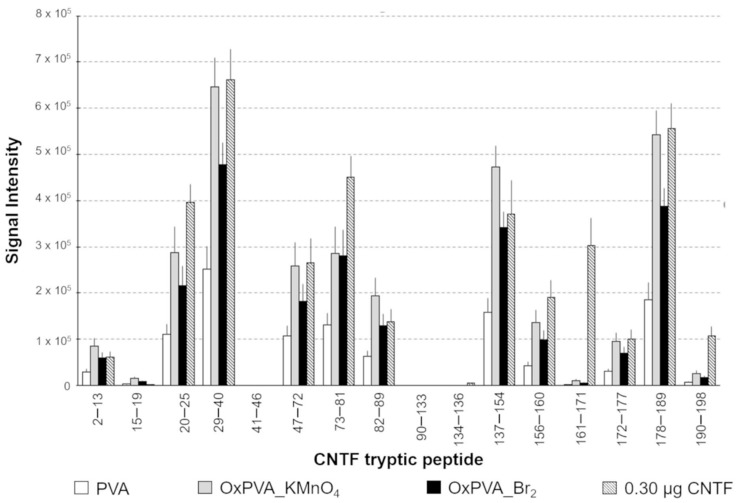
Neuroguide functionalization. Ultra high-performance liquid chromatography-mass spectrometry (UHPLC-MS) analysis of tryptic peptides derived from digestion of the ciliary neurotrophic factor (CNTF) bound in the luminal side of tubular scaffolds. The *y*-axis reports the peak area referred to the ion extracted by total ionic profile.

Nerve conduits made of OxPVA_Br_2_ were filled with a solution of CNTF to allow the absorption of the neurotrophic factor on the luminal surface of the tubular scaffolds. Neat PVA was also tested as a negative control characterized by low absorption capacity, whereas OxPVA_KMnO_4_ was considered as the positive control with improved protein-loading ability [12]. After factor binding stabilization by reduction of Schiff bases with sodium cyanoborohydride, the tubes were flushed with PBS and then filled with trypsin solution allowing the proteolysis of the small quantity of protein covalently linked inside the conduits. Tryptic peptides from nerve guides lumen were collected and subjected to analysis by UHPLC-MS. The histogram reported in Figure 7 shows tryptic peptides deriving from CNTF loaded into the lumen of neat PVA, OxPVA_KMnO_4_ and OxPVA_Br_2_ nerve conduits. The tryptic peptides derived from digestion of 0.30 µg of CNTF were also evaluated by UHPLC-MS as a reference for quantification of the neurotrophic factor.

Referring to the intensities of the peptide 29–40, the amounts of CNTF bound within the lumen of the three types of neuroguides were estimated as reported in Table 1. As demonstrated by relative quantification, the OxPVA_Br_2_ conduits were able to bind an amount of CNTF, which is comparable to the quantity detected in the lumen of OxPVA_KMnO_4_ conduits, with implemented loading ability in comparison with neat PVA.

## 4. Discussion

To date, surgical approaches for the treatment of peripheral nerve injuries with loss of substance are mainly based on autograft repair. Nevertheless, this strategy still presents important shortcomings, such as permanent co-morbidity at the donor site and limited sensorimotor functional recovery [1,2,3,4,5]. In this context, the development of bioengineered nerve conduits appears as a newsworthy strategy to implement current clinical PNI treatment [32]. Unlike autografting procedure, tubular scaffold implantation allows to avoid donor site morbidity, as well as guide sprouting axons while creating an isolated environment that protects the lesion from fibroblast and inflammatory cell invasion [33]. In particular, the ideal nerve guide should exhibit specific properties regarding not only high biocompatibility and the capacity to support directional axonal regeneration, but also enhanced bioactivity to mimic the native extracellular matrix in promoting Schwann cell adhesion, proliferation, migration and function [17].

Searching for this ideal neuroguide, tubular scaffolds made of blood vessels [34], muscles [35] or other biological materials (i.e., collagen [36], chitosan [37], silk fibroin [38]) have been deeply investigated, despite that they retain the risk of running into fibrosis, cell infiltration and loss of mechanical precision [32]. To overcome these problems, synthetic biodegradable materials ((i.e., poly(glycolic acid) (PGA), poly(lactic acid) (PLA) and their copolymer PLGA)) seem to represent the preferable choice, since they not only are biocompatible and low immunogenic, but also assure for the modulation of biodegradation rate and mechanical properties in order to customize the patient’s treatment [39]. Among synthetic polymers, which are under investigation for PNI repair, polyvinyl alcohol (PVA)-based tubular scaffolds have also been considered. Showing good biocompatibility, proper chemical and thermal stability, excellent tensile strength and flexibility, low toxicity, as well as high hydrophilicity, PVA was approved by Food and Drug Administration (FDA) for the manufacture of medical devices, including Salubridge™ and SaluTunnel™ nerve conduits [40]. Most important, mechanical behavior of PVA hydrogels can be modulated by variation of polymer molecular weight, cross-linking method and polymer solution concentration [41]. Nevertheless, a crucial limitation of PVA-based tubular scaffolds is represented by low biodegradability of the cross-linked polymer. Conversely, prior of cross-linking, immersion of PVA substrate in water leads to rapid sample dissolution with the formation of a clear, gelatinous mass [42]. Due to their non-biodegradable nature, commercial PVA conduits present high risks of infection, compression and scarring, which have limited their efficacy and safety for clinical application [40,43,44].

Based on the above considerations, we recently proposed the chemical oxidation of PVA by potassium permanganate in perchloric acid as a novel approach to obtain PVA-based scaffolds with ameliorated biological properties for tissue engineering purposes [12]. Interestingly, by replacing the 1% or 2% of hydroxyl groups with carbonyl groups into the polymer backbone, the swelling kinetics and protein absorption capacity of PVA hydrogel showed to increase along with the degree of oxidation. Noteworthy, subcutaneous implantation into the dorsal region of model mice allowed to demonstrate that oxidized PVA scaffolds induced no serious inflammatory reaction and mild lympho-monocytic infiltration of the connective tissue surrounding the implanted material, confirming the high biocompatibility and low immunogenicity of the hydrogels. Moreover, biodegradation rate of the oxidized polymer resulted in increasing proportionally to the degree of oxidation [12]. As a further step, we also employed 1% oxidized PVA with potassium permanganate (OxPVA_KMnO_4_) to fabricate bioabsorbable nerve conduits. Besides confirming biocompatibility and low immunogenicity, oxidized PVA-based tubular scaffolds demonstrated efficient regenerative potential when tested into a rat model of PNI [13]. Despite these promising results, an improvement of the technology may be still necessary, as chemical oxidation with potassium permanganate could lead to some alteration of polymer molecular weight. For this reason, we recently resorted to the use of a milder oxidizing agent—elementary bromine—to better preserve PVA physical properties. To the best of our knowledge, these are among the first efforts to implement PVA biological features to render it more suitable for nerve tissue engineering. Previously, chitosan/PVA nanofibers reinforced by carbon nanotubes only [45] or carbon nanotubes and bioactive glass particles [46] were manufactured to improve porosity, structural and biocompatibility properties of PVA-based scaffolds for neural regeneration. After an in vitro evaluation, these scaffolds showed to provide adequate mechanical properties and suitable environments for cell adhesion and growth. In addition, the fabrication of guidance conduits made of PVA loaded with functionalized multi-walled carbon nanotubes (MWCNTs) was successfully tested for in vivo peripheral nerve regeneration [47,48,49,50,51]. Investigated as tubular guides for neuro-muscular repair after an axonotmesis injury in rat sciatic nerves, PVA/MWCNT scaffolds were able to improve functional recovery and myelination of the regenerated nerve fibers without eliciting inflammatory response [51]. As a limit of this technology, there are some potential toxic concerns with the use of carbon nanotubes as scaffolds or drug delivery platforms [52].

In this work, we manufactured for the first time hollow nerve guidance conduits made of 1% oxidized PVA with bromine (OxPVA_Br_2_) by using the injection molding-technique and taking advantage of the physical freezing/thawing method for polymer cross-linking. This procedure avoids the use of any toxic chemicals, such as cross-linkers and initiators (i.e., glutaraldehyde [42]), allowing us to obtain PVA hydrogels with high cytocompatibility and thus excellent potential for biomedical applications [53]. This was confirmed by the cytotoxicity extract test, which provided insight to any potential issues with the local tissue response in the future perspective of in vivo implantation of nerve guides. Overall, the cytotoxicity response of SH-SY5Y and Schwann cells to oxidized PVA was demonstrated to be similar to the cytotoxic response of cells to known non-cytotoxic PVA, confirming that the prolonged dialysis of the polymer solution after oxidation ensured the elimination of any potentially toxic residual product derived from the chemical modification process.

Interestingly, OxPVA_KMnO_4_ and OxPVA_Br_2_ nerve guides turned out to be mechanically robust tubular constructs permitting excellent surgical handling, adequate suture holding and stretching, which are important properties for successful in vivo implant in PNI models. In particular, the neuroguides seemed to present good flexibility to maintain the lumen patency in response to limb flexion, while showing the adequate mechanical resistance to withstand the pressure of the surrounding tissues without collapsing. Important also, we confirmed that both KMnO_4_- and Br_2_-mediated oxidation can assure for increased biodegradability of scaffolds with respect to the native polymer, with OxPVA_Br_2_ appearing to be more sensible to enzymatic degradation than OxPVA_KMnO_4_. This suggests that the biodegradation rate of PVA-based conduits may be customized depending on nerve diameter, anatomical district and nerve depth, in order to adapt scaffolds to the remodeling process of the growing and regenerating nervous tissue.

Remarkably, as a key point for the production of bioactive tubular scaffolds, we decided to enhance the neurogenic potential of oxidized PVA conduits by the functionalization with the neurotrophic factor CNTF. As already stressed, we have previously demonstrated that PVA oxidation with potassium permanganate resulted in ameliorated protein loading ability of the polymer, also related to incremented swelling index [12]. As well known, the swelling ratio reflects the diffusional properties of a solute within a hydrogel [54]. This implies that swelling ratio represents an important measure for designing and optimizing a hydrogel as a drug delivery system. Given that, the swelling kinetics of OxPVA_Br_2_ and OxPVA_KMnO_4_ hydrogels was evaluated soon after the obtainment of the cross-linked polymers, in order to verify their potential to be used as drug delivery systems for CNTF controlled release. Referring to non-oxidized PVA, we confirmed that the new oxidation method with elementary bromine guaranteed for implemented swelling index in comparison with the non-modified polymer, with similar swelling kinetics curves of the two types of oxidized hydrogel. Strictly related to swelling behavior modification, the oxidation procedure also demonstrated to introduce higher porosity into PVA hydrogel, with the biological implication of improving the diffusion of specific growth factor solutions into the polymer structure. Noteworthy, the augmented porosity of oxidized hydrogels was related to an increased transparency of the corresponding neuroguides, with OxPVA_KMnO_4_ more transparent and more porous of OxPVA_Br_2_.

Based on these results, the final aim of the study was the functionalization of oxidized PVA-based tubular scaffolds with human CNTF. As previously reported, besides providing guidance, the controlled local delivery of therapeutic agents to the site of nerve injury results to be fundamental to accelerate the repair process [22]. To this end, a recent innovative technique based on electrospinning of polymer micro/nanofibers have proved to be very promising for the fabrication of drug releasing scaffolds incorporating growth factors that promote nerve regeneration [55,56,57].

Different growth factors (i.e., basic fibroblast growth factor (bFGF), NGF, BDNF, GDNF) have been tested to functionalize conduits made of natural materials [58,59,60], synthetic polymers [16,19,22,23,24] or a combination of the two [21,61,62,63]. In general, functionalized nerve guides demonstrated to promote superior functional recovery of injured nerves when tested in PNI animal models [19,21,22,23,24,58,59,60]. Regarding CNTF, its role in promoting nerve regeneration has been extensively investigated, as its local concentration gradient influences neuronal cell differentiation, neurite outgrowth, and the survival of motorneurons in vitro [18,64]. This neurotrophic factor was used together with BDNF to bi-functionalize silk fibroin electrospun nanofibers that promoted longer axonal growth in rat retinal ganglion cells when compared to non-functionalized nanofibers [65]. Similarly, CNTF and NGF were used together to fabricate bi-functionalized silk electrospun conduits by adding the factors into the spinning solution. These conduits were characterized in vitro, showing to promote glial cell migration, alignment and parallel axonal growth [66,67]. Again, collagen nerve conduits were filled with linear ordered collagen scaffolds combined with CNTF and bFGF, exhibiting effective capacity to stimulate facial nerve regeneration in minipigs [68]. Finally, considering CNTF-mediated functionalization of synthetic conduits, PLGA/chitosan nerve guides coated with CNTF were tested to repair 25-mm-long canine tibial nerve defects, showing to perform better than the non-functionalized guide and similarly to autologous nerve graft in assuring favorable therapeutic outcome [18].

Recently, our research group reported a preliminary study about the functionalization of PVA and OxPVA_KMnO_4_ cylinders with recombinant CNTF, which was modified through the linking to the transactivator transduction domain (TAT) to enhance protein cellular uptake for therapeutic advantage [25]. Differently from the aforementioned work, here we performed a more selective functionalization of neat and oxidized PVA-based conduits, by directly filling the lumen of tubular scaffolds rather than completely soaking them with the recombinant CNTF solution. Likely, the protein molecules were absorbed by diffusion into the pores of the hydrogel and/or by formation of Schiff-base interactions between carbonyl groups of the polymer and amino groups of the growth factor. In case of covalent binding formation, we implemented the experimental procedure by adding trypsin digestion treatment of the conduit lumen to recover bound CNTF, which was more difficult to be released. The functionalization experiment confirmed that the oxidation process implements the polymer ability to load protein molecules for drug-delivery purposes, with similar results for OxPVA_Br_2_ and OxPVA_KMnO_4_ regarding the estimated quantity of CNTF absorbed in the lumen of nerve guides. This could be ascribed to the fact that chemical oxidation introduces a) more carbonyl groups in the polymer backbone to mediate Schiff-base interactions with proteins and b) higher porosity to facilitate protein solution diffusion into the polymer chains.

## 5. Conclusions

In the present work, the fabrication and functionalization of 1% oxidized PVA-based nerve conduits were successfully performed. Collected data showed promising evidence about the possibility of using this technology to produce biocompatible and bioabsorbable nerve guides with implemented ability to load neurotrophic factors like CNTF to promote peripheral nerve regeneration. Here we carried out a preliminary investigation about OxPVA_Br_2_ tubular scaffold capacity to load protein molecules as efficiently as OxPVA_KMnO_4_ conduits. Further studies will compare more in detail the biomechanical properties of the two types of oxidized PVA hydrogels. Moreover, as a future perspective, more in-depth analyses will be necessary to test the effect of functionalized OxPVA_Br_2_ and OxPVA_KMnO_4_ conduits both in vitro, on neuronal cell culture assay and in vivo, through the surgical implantation into rat models of sciatic nerve injury. 

## Figures and Tables

**Table 1:** CNTF binding. Estimated amount of CNTF bound in the luminal surface of PVA- and oxidized PVA-based nerve guides.

Nerve Guidance Conduit	Area of Extracted IonP (29–40) m/z = 663.84 z = 2^+^	Bound CNTF (µg)
PVA	251,254 ± 50,751	0.11 ± 0.021
OxPVA_KMnO_4_	645,445 ± 63,984	0.29 ± 0.033
OxPVA_Br_2_	477,030 ± 48,103	0.22 ± 0.029

## Supplementary Materials

The following are available online at https://www.mdpi.com/1996-1944/12/12/1996/s1, Video S1: Mechanical features of PVA conduits, Video S2: Mechanical features of OxPVA_KMnO_4_ conduits, Video S3: Mechanical features of OxPVA_Br_2_ conduits.
